# Naringenin suppresses K562 human leukemia cell proliferation and ameliorates Adriamycin-induced oxidative damage in polymorphonuclear leukocytes

**DOI:** 10.3892/etm.2015.2185

**Published:** 2015-01-16

**Authors:** RUI-FANG LI, YING-QIAN FENG, JUN-HUI CHEN, LIN-TONG GE, SHU-YUAN XIAO, XUE-LAN ZUO

**Affiliations:** 1 Department of Neurology, Hubei Zhongshan Hospital, Wuhan, Hubei 430033, P.R. China; 2 Department of Pathology, Biological Sciences Division, The University of Chicago, Chicago, IL 60637, USA; 3 Department of Endocrinology, Weapon Industry 521 Hospital, Xi’an, Shaanxi 710065, P.R. China; 4 Department of Science and Education, Hubei Zhongshan Hospital, Wuhan, Hubei 430033, P.R. China; 5 Department of Hematology, Zhongnan Hospital of Wuhan University, Wuhan, Hubei 430071, P.R. China

**Keywords:** naringenin, cell line, K562, gene, p21, gene, p53, cell cycle, apoptosis

## Abstract

Treatments for leukemia remain unsatisfactory. Conventional chemotherapy agents that aim to kill tumor cells may also damage normal cells and thus result in severe side-effects. Naringenin, a natural polyphenolic compound with antioxidant effects, has been revealed to have significant antitumor effects with low toxicity in preliminary studies. Thus, it is considered as one of the most promising flavonoids in the treatment of leukemia. In the present study, the effects of naringenin on the K562 human leukemia cell line and the underlying mechanisms were explored *in vitro*. In addition, human peripheral blood polymorphonuclear leukocytes (PMNs) were used as a normal control in order to evaluate the effects of naringenin on normal granulocytes and in the mediation of Adriamycin (ADM)-induced oxidative damage. The results revealed that K562 proliferation was significantly inhibited by naringenin in a time- and concentration-dependent manner; however, minimal cytotoxic effects were observed in PMNs when naringenin was used at concentrations <400 μmol/l. Morphological changes indicative of apoptosis were observed in naringenin-treated K562 cells. Flow cytometric analysis indicated that the K562 cells were arrested in the G_0_/G_1_ phase of the cell cycle with a significantly upregulated rate of apoptosis. Furthermore, in the naringenin-treated K562 cells, the labeling index of proliferating cell nuclear antigen was observed to be increased by immunochemical staining, the mRNA and protein expression levels of p21/WAF1 were strongly upregulated in reverse transcription-polymerase chain reaction and western blot analyses, whereas p53 gene expression was not significantly changed. In PMNs to which naringenin (50~80 μmol/l) was added 1 h subsequent to ADM, the cell damage induced by ADM was significantly reduced, coincident with reductions in the levels of reactive oxygen species (ROS) and malondialdehyde (MDA) and increases in the activity of superoxide dismutase and glutathione peroxidase. However, the cytotoxic effect of ADM in K562 cells was not significantly altered by naringenin, and the oxidative stress indices in K562 cells remained stable. In conclusion, the present study revealed the promising value of naringenin in leukemia treatment. Naringenin demonstrated a significant inhibitory effect on the growth of K562 cells but not on normal PMNs. Furthermore, naringenin protected PMNs from ADM-induced oxidative damage at low concentrations. Cell cycle arrest and apoptosis-inducing effects, achieved through p53-independent p21/WAF1 upregulation, are likely to be the mechanism of the antileukemic effects of naringenin, and the protective effect against ADM chemotherapy-induced damage in PMNs may be due to the antioxidant capability of this agent at low concentrations.

## Introduction

Leukemia, a malignancy that originates in hematopoietic stem cells, now ranks as the seventh most common malignant tumor in China, accounts for a population-standardized incidence rate of 2.76/10 million, and is one of the most significant causes of mortality in the Chinese population (1).

The overall efficacy of leukemia treatment remains unsatisfactory, despite progress in basic research of its pathogenesis and improvements in clinical therapy. Chemotherapy is one of the most important treatments for leukemia. However, conventional chemotherapy agents, such as Adriamycin (ADM), which kill tumor cells by mediating cell apoptosis or necrosis, act on normal cells in the same manner and are thought to result in serious side-effects; therefore, the application of many such agents is limited (2). The search for new antileukemic agents with high efficiency and low toxicity has become an important task. It has been revealed that certain natural medicines, for example, flavonoids or natural polyphenolic compounds, have extensive pharmacological effects with low toxicity (3,4). Thus, they may be expected to have a role in the treatment of leukemia and have attracted an increasing amount of attention. Naringenin, a widely distributed natural flavonoid that is one of the most abundant flavonoids in citrus fruit, is formed from its precursor, naringin, by removal of part of the molecule by gut bacteria following oral ingestion (5,6). Naringenin exhibits extensive pharmacological effects, including free radical-scavenging activity and antioxidant, antiproliferative and anti-atherogenic effects (7–12). Preliminary studies suggest that naringenin may play an important role in cancer prevention and treatment, such as by inducing cell cycle arrest or apoptosis, reversing drug resistance, inhibiting subcellular signal transduction and promoting DNA repair (13–15). Naringenin has exhibited antitumor activity against a variety of tumor cells in previous studies (14–17). Chen *et al* (18) reported that naringenin effectively inhibited the proliferation of the leukemia cell line HL-60, while having little cytotoxicity in mature THP-1 monocytes and normal peripheral blood polymorphonuclear leukocytes (PMNs), which suggests that naringenin may be promising in leukemia treatment. However, the antileukemic effects and antitumor mechanisms of naringenin are yet to be understood and contradictions in existing reports remain to be clarified (19).

The human leukemia cell line K562 was established by Lozzio and Lozzio (20) from cells obtained from a patient in the blastic phase of chronic granulocytic leukemia. The phenotype of the cell line includes the immunological markers CD3 (−), CD13 (+), CD19 (−), CD34 (−), CD41 (+), CD42 (+), CD71 (+) and CD235a (+), and the carrying of the BCR/ABL fusion gene, which promotes cell growth, inhibits apoptosis and causes defects of DNA repair (21). K562 cells are commonly used in cell culture for studies of drug effects on leukemia. In the present study, the effects of naringenin on the human leukemia cell line K562 *in vitro* and the underlying mechanisms were explored. Moreover, human peripheral blood PMNs were cultured as normal cells of the control group so that the effect of naringenin on normal granulocytes and its ability to ameliorate ADM-induced oxidative damage could be evaluated. The aim of the study was to assess the value of naringenin in leukemia treatment in order to explore new methods for the therapy of leukemia.

## Materials and methods

### Reagents

Naringenin, Wright-Giemsa stain and Hoechst 33258 stain were obtained from Sigma-Aldrich (St. Louis, MO, USA). Naringenin was of >98% purity, dissolved in DMSO at a concentration of 400 mmol/l and stored at −20°C. ADM was from Pharmacia & Upjohn (Peapack, NJ, USA). TRIzol, low melting point agarose and horseradish peroxidase-conjugated goat anti-mouse polyclonal immunoglobulin G (IgG) secondary antibody were from Promega (Madison, WI, USA). RevertAid™ First Strand cDNA Synthesis kit, Moloney murine leukemia virus (M-MLV) reverse transcriptase and Taq DNA polymerase were from MBI Fermentas (Burlington, CA). 3-[4,5-Dimethylthiazol-2-yl]-2,5-diphenyltetrazolium bromide (MTT) was from Sigma-Aldrich. RPMI-1640 medium, fetal bovine serum (FBS) and trypsin-ethylenediamine tetraacetic acid (EDTA) were from Hyclone (Thermo Scientific, Logan, UT, USA). Mouse anti-human proliferating cell nuclear antigen (PCNA) monoclonal antibody, horseradish peroxidase (HRP)-conjugated goat anti-mouse IgG antibody, 3,3′-diaminobenzidine tetrahydrochloride and H_2_O_2_ were from Santa Cruz Biotechnology, Inc. (Santa Cruz, CA, USA). Normal goat serum was from Zhongshan Bio-Tech Co., Ltd. (Beijing, China). Protein Detector™ Western Blot kit was from KPL, Inc. (Gaithersburg, MD, USA). Polyvinylidene fluoride (PVDF) membranes were from Millipore (Bedford, MA, USA). The protein marker was from MBI Fermentas. MDA, superoxide dismutase (SOD), ROS and glutathione peroxidase (GSH-Px) assay kits were from Jiancheng Bioengineering Research Institute (Nanjing, China). EDTA, penicillin and streptomycin were from Gibco (Invitrogen Life Technologies, Carlsbad, CA, USA).

### Cell culture

The human K562 cell line was obtained from the China Center for Type Culture Collection (CCTCC) of Wuhan University (Wuhan, China), and was cultured in RPMI-1640 medium containing 10% FBS, 1 mmol/l glutamine and 10 U/l penicillin and streptomycin. Human PMNs were isolated from the citrate-anticoagulated peripheral blood of healthy donors by Polymorphprep centrifugation techniques as described previously (22). The purity of human PMNs was >95% as estimated by Wright-Giemsa staining. PMNs were suspended in PBS containing 1 mmol/l CaCl_2_ and 1 mmol/l MgSO_4_. All cells were maintained in a humidified 5% CO_2_ atmosphere at 37°C. Cells were split at a ratio of 1:2 once they reached 70–90% confluence. Generally, the K562 cells grew into a monolayer within 2–3 days, and were continually cultured for 2 to 3 passages for use in the experiments. Written approval for the derivation, culture and experimental use of the PMNs was obtained from the Ethics Committee, Zhongnan Hospital of Wuhan University (Wuhan, China).

### MTT assay

Cell viability was determined using an MTT assay. Briefly, single cell suspensions of K562 cells and PMNs were seeded onto 96-well plates at a density of 1×10^5^/well and incubated for 24, 48 or 72 h at 37°C in a 5% CO_2_ culture incubator. Cells were treated with naringenin at final concentrations of 0, 50, 100, 200, 400 and 800 μmol/l respectively, with five wells for each group. After incubation for 24 h, 20 μl MTT reagent (5 mg/ml) was added to each well and the cells were incubated for 4 h. Then, the formazan precipitate was dissolved in 150 μl DMSO and the absorbance value was measured using a microplate reader (ELx808; BioTek, Winooski, VT, USA) at a wavelength of 570 nm. The cell proliferation inhibition rate (%) was calculated as: (A_0_−A)/A_0_ × 100%, where A represents the mean absorbance value of the sample cells, and A_0_ represents the mean absorbance value of the control sample.

### Immunocytochemical testing and labeling index (LI) calculation

Immunocytochemical staining was carried out as described previously (23). Briefly, cells were fixed with methanol at −20°C for 15 min and then were washed with PBS. The fixed cells were permeabilized with 1% NP-40 and blocked with 10% normal goat serum, followed by incubation with appropriately diluted (1:100) PCNA primary antibody for 24 h at 4°C. The cells were washed with PBS and exposed to the horseradish peroxidase (HRP)-conjugated goat anti-mouse IgG antibody (1:1,000) for 30 min at room temperature. Staining was performed as a 5 min exposure to 3,3′-diaminobenzidine tetrahydrochloride/H_2_O_2_, which formed a brown precipitate on the labeled cells. Stains without the primary antibody were used as negative controls. The presence of proteins of interest was examined under a light microscope (CKX41; Olympus Corporation, Tokyo, Japan). The LI was obtained by counting the number of positive cells per 100 cultured cells in 10 fields of vision.

### Analysis of apoptotic cells

#### Light microscope observation

K562 cells with Wright-Giemsa staining and Hoechst 33258 staining were observed under an inverted microscope (CKX41; Olympus Corporation) at ×400 magnification.

#### Immunofluorescent staining

Chromatin condensation was detected by nuclear staining with Hoechst 33258. Briefly, cells were fixed with 2% formaldehyde for 10 min, stained with phosphate-buffered saline (PBS)/0.1% Triton-X 100/10 μM Hoechst 33258 for 5 min, then were visualized by inverted fluorescence microscopy (Leica DM IRB; Leica Microsystems GmbH, Wetzlar, Germany). Apoptotic cells were stained bright blue due to chromatin condensation.

#### Ultrastructure observation by transmission electron microscopy (TEM)

Briefly, 1×10^5^/ml K562 cells were seeded and incubated for 24 h at 37°C in a 5% CO_2_ culture incubator. Naringenin was added to attain various final concentrations as previously described. After incubation for 48 h, cells were harvested with serum-free Dulbecco’s modified Eagle’s medium (DMEM) and fixed in 3% glutaraldehyde for 4 h. Then, the cells were washed twice with PBS, fixed in 1% osmic acid for 1 h, dehydrated and embedded. The 70-nm slices were prepared and observed under a transmission electron microscope (JEM-2200FS; Jeol, Ltd., Tokyo, JP) after staining with uranyl acetate and lead citrate.

### Flow cytometric analysis of the cell cycle

K562 cells at a density of 1×10^5^/ml were seeded in 6-well dishes, and naringenin was added to a final concentration of 50, 100, 200, 400 and 800 μmol/l, respectively. 0 μmol/l naringenin was set as the control. After 24, 48 and 72 h of culturing, cells were trypsinized, washed twice with cold PBS and centrifuged at 16 × g for 3 min. The pellet was resuspended in 50 ml cold PBS and 450 μl cold methanol for 1 h at 4°C. The cells were centrifuged at 1,000 × g for 5 min, and the pellet was washed twice with cold PBS, suspended in 500 μl PBS, and incubated with 5 ml RNase (20 μg/ml final concentration) for 30 min. The cells were chilled over ice for 10 min and stained with propidium iodide (50 μg/ml final concentration) for 1 h and analyzed by flow cytometry (FACScan; Becton Dickinson, Franklin Lakes, NJ, USA).

### Semi-quantitative reverse transcription polymerase chain reaction (RT-PCR) for p53 and p21/WAF1

After 24, 48 and 72 h of culture with 400 μmol/l naringenin, total RNA was extracted from each group using TRIzol reagent following the manufacturer’s instructions and quantitated by absorbance at 260 nm. For the RT-PCR, the RevertAid™ First Strand cDNA Synthesis kit was used with a total RT reaction volume of 10 μl. The reaction temperature was 30°C for 10 min, 42°C for 20 min and 45°C for 30 min. For the PCR stage, the total reaction volume was 50 μl. PCR was performed in a GeneAmp PCR system 2400 (Perkin Elmer, Waltham, MA, USA). Primers for p21 were, forward: 5′-GATGTCCGTCAGAACCCATG-3 and reverse: 5′-CCACATGGTCTTCCTCTGCTG-3′, with an expected fragment size of 316 bp. For p53, the forward primer was 5′-GTCTGTGACTTGCACGTACT-3′ and the reverse 5′-CAGTCAGAGCCAACCTCAGG-3′, with an expected fragment size of 326 bp. β-actin was used as the internal standard reference, with forward primer 5′-GTGGGGCGCCCCAGGCACCA-3′, reverse primer 5′-CTCCTTAATGCACGCACGATTTC-3′, and an expected 500-bp band. After cDNA (3 μl) and specific primers were added to the master mix, PCR was conducted with initial denaturation at 94°C for 5 min, followed by 24 cycles of denaturation at 94°C for 30 sec, annealing at 50°C for 30 sec and extension at 72°C for 30 sec. A 5 μl sample of the PCR products was visualized by electrophoresis on 1% agarose gel stained with ethidium bromide and quantitated by densitometry using the ImageMaster VDS system and associated software (GE Healthcare Life Sciences, Uppsala, Sweden).

### Western blot analysis of p53 and p21/WAF1 proteins

K562 cells were seeded in 6-well plates at a density of 1.0×10^5^/ml and divided into four groups. Naringenin was added to each group to attain a final concentration of 400 μmol/l. Then, cells in each group were cultured for 24, 48 and 72 h respectively; a 0 h group was used as a control. Cells were collected by centrifuging at 1,000 × g for 3 min and washed with PBS. The cell pellets were resuspended in sodium dodecyl sulfate (SDS) sample buffer (62.5 mmol/l Tris-HCl pH 6.8, 2% SDS, 10% glycerol, 50 mmol/l dithiothreitol, 0.1% bromphenol blue), incubated for 5 min at 95°C, cooled on ice for 5 min and stored at −20°C until further use. Cell lysates were subjected to SDS-polyacrylamide gel electrophoresis (PAGE) using 10% polyacrylamide gels and transferred to PVDF membranes using a semidry electroblot chamber. Proteins in the gel were assessed by Coomassie brilliant blue staining. Membranes were blocked in Tris-buffered saline pH 7.4 containing 0.1% Tween-20 and 5% bovine serum albumin for 1 h at room temperature. Incubations with the primary antibodies: Monoclonal mouse anti-human p53 and monoclonal mouse anti-human p21/WAF1 (GE Healthcare, Piscataway, NJ, USA), were carried out at 4°C overnight using antibody dilutions as recommended by the manufacturer in Tris-buffered saline pH 7.4, 0.1% Tween-20. Following 1 h of incubation with peroxidase-conjugated goat anti-mouse polyclonal IgG secondary antibody (dilution 1:2,000) at room temperature, proteins were detected by the electrogenerated chemiluminescence method according to the manufacturer’s instructions (GE Healthcare). As a loading control, blots were assayed against β-actin.

### Attenuation of ADM chemotherapeutic injury by low-dose naringenin

The MTT assay was used to test cell viability, as described earlier. The concentration of ADM was set in five groups as 0.75, 1.5, 3.0, 6.0 and 12.0 μmol/l, respectively, with three wells for each group. After culturing for 48 h, the half maximal (50%) inhibitory concentration (IC_50_), that is, the working concentration of ADM, was determined for further experiments. Then five different groups were established: Control, naringenin, ADM, post 1 h (naringenin addition 1 h after co-culturing with ADM) and post 24 h (naringenin addition 24 h after co-culturing with ADM) groups. Final concentrations of naringenin of 5, 10, 20, 40 and 80 μmol/l were used in each group. In the control group, an equal volume of DMSO was used and its final content was <5% by volume.

### Detection of ROS, SOD, MDA and GSH-Px

Cell lysates of K562 cells and PMNs were generated as previously described (24). The content of MDA and activities of SOD and GSH-Px were determined using assay kits, following the manufacturer’s instructions.

### Statistical analysis

Data were analyzed for significance with an unpaired t-test and analysis of variance test. Statistical software SPSS 13.0 (SPSS, Inc., Chicago, IL, USA) was used in the analysis. P<0.05 was considered to indicate a statistically significant difference.

## Results

### Effect of naringenin on the proliferation of K562 cells

**Figure 1:**
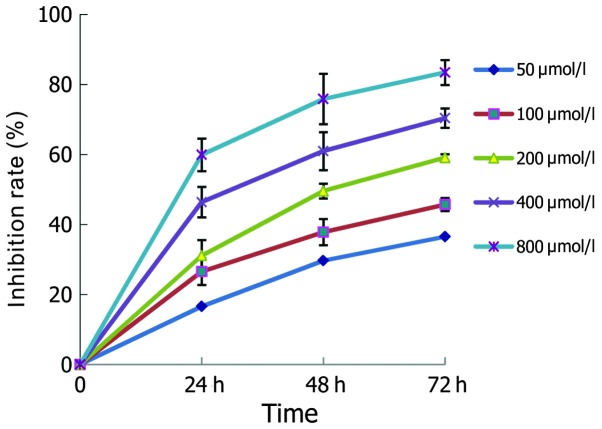
Growth inhibitory effect of naringenin on K562 cells.

**Figure 2:**
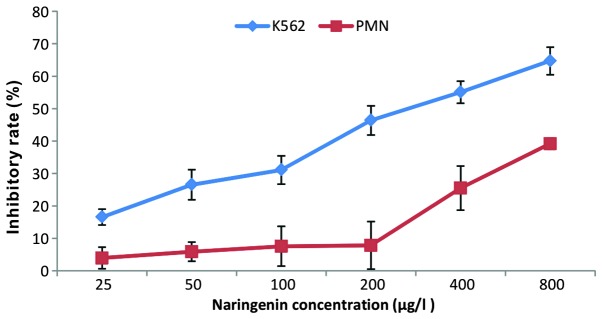
Effects of naringenin on the growth of K562 cells and PMNs (48 h). PMN, polymorphonuclear leukocyte.

As shown in Fig. 1, the growth of K562 cells was inhibited by naringenin in concentration- and time-dependent manner. However, naringenin at low concentrations did not show a cytotoxic effect on normal PMNs until its concentration was increased to 400 μmol/l (Fig. 2).

### Effects of naringenin on cell morphology

**Figure 3:**
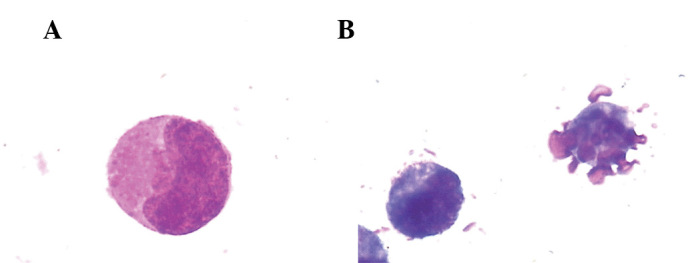
Morphologic observation of K562 cells with Wright-Giemsa staining. Cell morphology of (A) K562 cells in the control group and (B) K562 cells in the naringenin-treated group, which indicates the formation of apoptotic bodies. Magnification, ×1,000.

**Figure 4:**
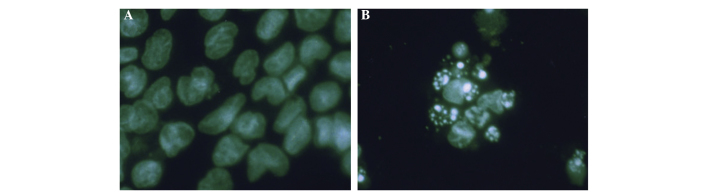
Morphological changes in K562 cells following naringenin treatment as revealed by confocal microscopy and Hoechst 33258 fluorescence staining. (A) K562 cells in the control group. (B) K562 cells treated with 400 μmol/l naringenin treatment for 24 h, which indicates morphological changes typical of apoptosis and the formation of apoptotic bodies. Magnification, ×400.

**Figure 5:**
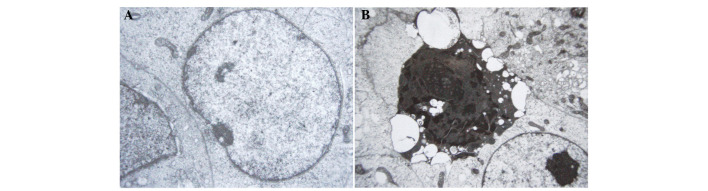
Ultrastructural changes of K652 cells with TEM observation. (A) Ultrastructure of K562 cells in the control group. TEM shows a smooth cell body, evenly dispersed chromatin and complete membrane and nuclear membrane. (B) K562 cells treated with 400 μmol/l of naringenin for 24 h displaying morphological changes typical of apoptosis, including cell nuclear condensation, nuclear membrane rupture, the formation of clumps along the nuclear membrane or crescent formation, and cytoplasm blistering. Magnification, ×15,000. TEM, transmission electron microscopy.

When observed under an inverted microscope (CKX41; Olympus, Tokyo, Japan), the proliferation of K562 cells was inhibited following treatment with naringenin. Morphologic changes typical of apoptosis and the formation of apoptotic bodies were observed by Wright-Giemsa staining (Fig. 3). Immunofluorescent staining results are shown in Fig. 4. Cells in the control group presented abundant cytoplasm, complete membrane integrity and homogeneous nuclear mass. However, cells in the treatment groups exhibited shrinking membranes, smaller nuclei, concentrated aggregates, and variously sized fragments formed by nuclear fragmentation. Moreover, polygonal, circular or petal-shaped apoptotic bodies, which represent the typical morphology of apoptosis, were observed. Fragmentation of dead cells occurred when the naringenin concentration was increased to the highest level (800 μmol/l). TEM observation revealed the ultrastructural changes of K652 cells following naringenin treatment. As is shown in Fig. 5, cells in the control group exhibited large nuclei, uniform nuclear chromatin, a small quantity of heterochromatin and prominent nucleoli. However, in the naringenin-treated group, the cells presented massive aggregation of nuclear chromatin, or fragmented pieces that were coated with nuclear membrane, which indicated apoptotic morphological change.

### Immunocytochemical staining of PCNA and LI results

It was observed that the positive expression of PCNA was mainly located in the nucleus and was visible as brown staining or particles in the nucleus or even part of the cytoplasm. LI calculations indicated that the positive expression rate of PCNA in the control group was significantly higher than that in the naringenin-treated groups [33.14±3.6 vs. 19.67±2.21 (400 μmol/l, 24 h naringenin); P<0.05].

### Flow cytometric analysis of the cell cycle

As shown in Table I, after 24 h of treatment with naringenin, the proportion of K562 cells in the G_0_/G_1_ phase increased whereas the proportion in the S phase decreased, which indicates that naringenin probably contributed to G_0_/G_1_ phase arrest of the K562 cells.

### Semi-quantitative RT-PCR testing of the expression of p53 and p21/WAF1

**Figure 6:**
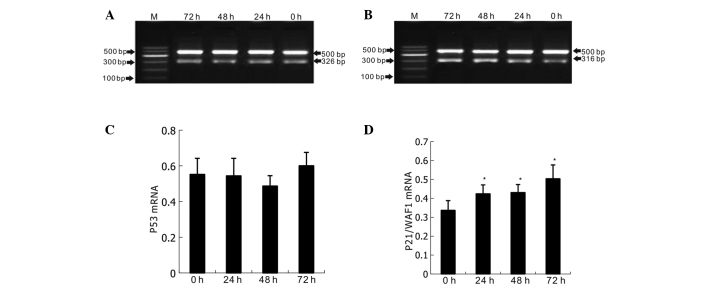
Semi-quantitative RT-PCR testing of p53 and p21/WAF1 expression in K562 cells with 400 μmol/l naringenin treatment. (A) and (B) electrophoresis of RT-PCR for p53 and p21 mRNA, respectively. M represents marker; bands from bottom to top indicate 100, 200, 300, 400, 500 and 600 bp in the ladder, respectively. β-actin (500 bp band) was used as an internal standard. The target gene fragment of p53 is 326 bp and of p21/WAF1 is 316 bp. (C) and (D) Bar charts of the relative content analysis of p53 and p21/WAF1 mRNA, respectively. Naringenin was used at a dose of 400 μmol/l. ^*^P<0.05 vs. 0 h.

As shown in Fig. 6, when naringenin at a concentration of 400 μmol/l was added and the K562 cells were cultured for 24, 48 and 72, significant increases in the expression levels of p21/WAF1 mRNA (P<0.05) were detected, whereas p53 mRNA expression remained stable (P>0.05). The results also indicated that p21/WAF1 mRNA was upregulated in a time-dependent manner, and began as early as 24 h post naringenin treatment.

### Western blot analysis of p53 and p21/WAF1 proteins

**Figure 7:**
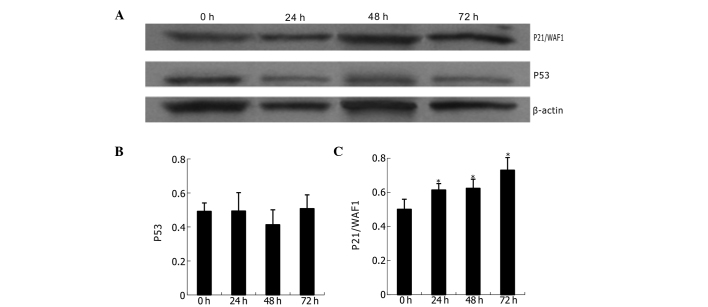
Western blots of p53 and p21/WAF1 protein in K562 cells treated with naringenin. (A) Western blot analysis of p53 and p21/WAF1 protein expression in K562 cells; β-actin was used as internal standard. (B) and (C) Bar charts of the relative content analysis of p53 and p21/WAF1, respectively, which were calculated by comparing the target gene to the internal reference β-actin. Naringenin was used at a dose of 400 μmol/l. ^*^P<0.05 vs. 0 h.

The expression of p53 and p21/WAF1 proteins is shown in Fig. 7. Compared with untreated K562 cells, the naringenin-treated cells (400 μmol/l) revealed statistically significant upregulation of p21/WAF1 but not of p53 at different time points (24, 48 and 72 h). Analysis indicates a time-dependent and p53-independent mode of p21/WAF1 upregulation.

### Modulating effect of low-dose naringenin on ADM chemotherapeutic injury in PMNs and K562 cells

The inhibition rates of various concentrations of ADM (0.75, 1.5, 3.0, 6.0 and 12.0 μmol/l) were as follows: 30.2±2.9, 44.9±3.8, 56.7±4.2, 66.6±3.7 and 75.4±5.3%, respectively. According to these results, the IC_50_ was calculated to be 2.05±0.24 μmol/l. Therefore, a working concentration of 2 μmol/l of ADM was used for further experiments.

**Figure 8:**
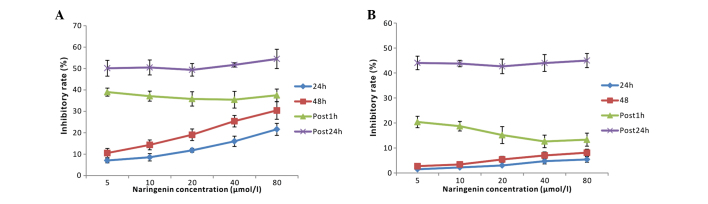
Efficacy of naringenin in modulating the chemotherapeutic injury exerted by ADM on PMNs and K562 cells. Effects on (A) K562 cells and (B) PMNs. ADM, Adriamycin; PMN, polymorphonuclear leukocyte.

Naringenin exhibited a small suppressive effect (<8.1%, P>0.05) on normal human PMNs, despite its 7–35% inhibitory rate in K562 cells at low concentrations (5–80 μmol/l). As shown in Fig. 8, neither post 1 h nor post 24 h naringenin addition significantly attenuated the cytotoxic effect of ADM on K562 cells. However, for PMNs, naringenin that was added post 1 h, but not post 24 h ADM treatment, significantly decreased the injury induced by the ADM chemotherapy, accounting for an inhibitory rate of 38.18±2.43%.

### ROS, SOD, MDA and GSH-Px testing during the modulation of ADM chemotherapeutic injury by naringenin

The effects of naringenin on the ADM-induced changes in antioxidant enzyme activities in K562 cells and PMNs are shown in Table II. The results revealed that ADM significantly reduced the enzymatic activity of GSH-Px and SOD in the K562 cells and PMNs. When naringenin (20 or 40 μmol/l) was added 1 h post ADM treatment, the two intracellular enzymes were significantly upregulated in PMNs but not in K562 cells; however, no upregulating effect was observed in the post 24 h group. Similarly, ADM alone caused increases in the levels of the intracellular oxidation products of ROS and MDA in K562 cells and PMNs, whereas low-dose naringenin in the post 1 h group benefitted PMNs but not K562 cells by the reduction of intracellular ROS and MDA levels (Table II). These data indicate that low-dose naringenin ameliorates the chemotherapeutic injury induced by ADM in normal PMNs without weakening the cytotoxic effect of ADM on K562 tumor cells through its antioxidant effect.

## Discussion

Natural medicine has become a popular topic in hematological research in recent years due to the high antineoplastic efficiency and low toxicity of certain natural compounds. In the current study, the effects and mechanisms of the natural flavonoid naringenin on the human leukemia cell line K562 as well as on normal human peripheral PMNs were investigated. In addition, the effects of low-dose naringenin on ADM-induced injury in K562 cells and PMNs were evaluated. In order to clarify the dose-response relationship, naringenin concentrations ranging from 50 to 800 μmol/l were tested with the aim of rapidly obtaining growth inhibition while concurrently avoiding the appearance of cell necrosis over a certain period of time. The results indicate that naringenin exerted significant cell proliferation suppressing effects on K562 cells in a dose- and time-dependent manner. Microscopic observation also revealed that significant necrosis, which is indicative of cytotoxicity, occurred when the concentration of naringenin was increased to 800 μmol/l.

PCNA, a 36-kDa non-histone nucleoprotein that is synthesized at the G_1_ and S phases of the cell cycle and is directly involved in DNA synthesis by acting as a cofactor of DNA polymerase δ, is a one of the most common indicators of tumor proliferation kinetics (25). Gene-mediated regulation of PCNA is an important mechanism by which cell proliferation is modulated (26). In the current study, it was observed that the downregulation of PCNA was coincident with the inhibition of proliferation in K562 cells, which revealed the cell growth suppression efficiency of naringenin in K562 cells. It was also found that <100 μmol/l naringenin exhibited a moderate inhibition rate of <8.1% in PMNs (P>0.05), which was far less than that in K562 cells. However, statistically significant growth inhibition of PMNs appeared when the naringenin concentration was increased to 400 μmol/l. These data suggest that naringenin at low concentrations inhibited the growth of K562 cells but had little effect on normal human neutrophils. This phenomenon reveals the advantage of this natural medicine in antitumor application. Although the exact mechanism remains unclear, it may be attributed to the higher metabolic rate of tumor cells compared with that of normal cells.

Abnormality of apoptosis and cell cycle control, which widely exist in tumor cells and tissues, are important in tumor progress and occurrence. As a result, strategies targeting apoptosis or cell cycle control have been critically significant in the development of new anticancer agents. In the present study, morphological changes typical of apoptosis were observed by Wright-Giemsa staining, immunofluorescent staining, and TEM observation. However, cytotoxicity rapidly manifested as necrosis when the concentration of naringenin was increased to 800 μmol. A quick and accurate analysis of the cell cycle and apoptosis may be achieved by quantifying cell chromosomes via flow cytometric techniques. The results of the present study reveal that K562 cells were arrested in the G_0_/G_1_ phase by naringenin in a concentration-dependent manner. The foregoing analysis indicates that inducing apoptosis and cell cycle arrest may be a key mechanism by which naringenin inhibits K562 cell proliferation. Moreover, cytotoxicity appeared when naringenin was used at a higher concentration. Sanderson *et al* (17) reported an inhibitory effect of naringenin on the human adrenocortical carcinoma cell line H295R with an IC_50_ of 85 μmol/l, and found that cytotoxicity occurred when the concentration exceeded 1,000 μmol/l. Previous literature (18) suggests that naringenin induces apoptosis through a caspase-3/CPP32 apoptotic pathway in HL-60 cells, and has cytotoxic effects at high doses.

Tumorigenesis is a multi-factorial, multi-stage and cumulative process that is essentially characterized by the activation of oncogenes and inactivation of tumor suppressor genes, the reduction of apoptosis and increase of proliferation, cell dedifferentiation abnormalities and numerous other aspects of cell life dysregulation, which eventually lead to uncontrolled growth of cells (27,28). However, almost all the dysregulation of oncogenes, tumor suppressor genes and other cell regulation factors eventually converge as abnormalities of cell cycle regulation. Therefore, tumors are, in essence, a type of cell cycle disease. Cell cycle progression is accurately regulated by complicated network system that consist of cyclins-CDKs-CKIs and oncogenes/tumor suppressor genes. For example, there is evidence that p53 and p21 genes play extremely vital roles in regulating the cell cycle (29). The p21 protein binds to and inhibits the activity of cyclin-CDK2, −CDK1, and −CDK4/6 complexes, and thus functions as a regulator of cell cycle progression at the G_1_ and S phases (30). p21/WAF1 can also interact with PCNA, a DNA polymerase accessory factor, and plays a regulatory role in S phase DNA replication and DNA damage repair. p21/WAF1 is also known as wild-type p53-activated fragment and acts through inducing the activity of tumor suppressor gene p53 (31). Anything that impedes the activation of p53 and p21/WAF1 will result in dysfunction of the negative regulatory factors on the cell cycle protein complexes, finally leading to the loss of normal control of the cell cycle and thus malignant proliferation. In the present study, it was found that the proliferation of K562 cells was inhibited by naringenin and cell growth was arrested in the G_0_/G_1_ phase of the cell cycle. In order to elucidate the underlying mechanism, the expression of p53 and p21/WAF1 was examined by RT-PCR and western blotting. It was found that p21/WAF1 was expressed at low levels in the control group, but the expression level increased significantly in a time-dependent manner following naringenin treatment, which is similar to the findings reported by Panno *et al* (32) in research concerning the breast carcinoma cell line MCF-7. Therefore, it is speculated that the induction of p21/WAF1 expression is likely to be an important mechanism for the antiproliferative effect of naringenin on K562 cells. However, p21 may inhibit apoptosis and does not induce cell death on its own (33).

p21 is located downstream of the p53 gene. It has been confirmed that p21 expression is mediated by p53-dependent and p53-independent pathways (34–36). In the former pathway, the expression of p21/WAF1 is tightly controlled by the tumor suppressor protein p53, through which this protein mediates p53-dependent cell cycle G_1_ phase arrest in response to a variety of stress stimuli (37), including DNA damage or even cancer cells themselves. However, p21/WAF1 can also be activated directly by mitogens instead of p53 if cell proliferation is highly active, which is referred to as a p53-independent pathway. The current study indicates that the p53 gene was expressed in the control and therapy groups, and that the expression level remained stable at various time points and naringenin concentrations, which indicates that naringenin functions in a p53-independent manner. In a study concerning seven kinds of natural flavonoids with similar molecular structures (18), it was reported that the antitumor effects of these agents were dependent on the manner of apoptosis induction in a p21- but not p53-dependent pathway. Kanno *et al* (16) concur with this antitumor mechanism of flavonoids. Apoptosis is gene-encoded programmed cell death, has specific biological characteristics and is coincident and interrelated with the cell cycle (35). Conclusions can be drawn from previous data and analysis that the upregulation of p21/WAF1 may be the underlying mechanism by which naringenin inhibits K562 tumor cell growth. However, whether there are other mechanisms involved in this process is yet to be clarified.

As has been demonstrated by the results of the present study, naringenin has modest cytotoxicity to normal PMNs. Naringenin, similar to other natural flavonoids, has also been demonstrated to be an antioxidant that is characterized by its ability to scavenge free radicals (38,39). The present study further explored whether low concentrations of naringenin (5–80 μmol/l) could generate an antioxidative effect so as to alleviate the toxicity of ADM to normal cells. ADM, one of the most potent anthracyclines with a wide spectrum of anticancer activity, has been shown to be effective in the treatment of acute leukemia, lymphoma and several solid tumors (40). However, side-effects of the agent are also evident as it kills normal cells in chemotherapy. In the present study, when naringenin was added to PMNs 1 h after the addition of ADM, the chemotherapeutic injury to the PMNs was significantly decreased, whereas there was little effect on K562 cells to which exactly the same drug treatments were administered. It was also observed that intracellular antioxidant enzyme activities increased and oxidation products decreased in PMNs but not K562 cells, when naringenin was added 1 h after ADM treatment; thus, the effects of the ADM treatment in strongly upregulating the oxidation products of ROS and MDA and downregulating the activities of the antioxidant enzymes GSH-Px and SOD were attenuated. This reveals that the addition of naringenin can help to reduce chemotherapeutic injury to normal cells without decreasing the cytotoxicity of ADM to K562 cells, as ADM may kill tumor cells by a mechanism other than by the induction of oxidative damage. These results suggest that low-dose naringenin helps to ameliorate the ADM-induced chemotherapeutic injury of normal blood cells without weakening its cytotoxic effect on tumor cells via oxidative modulating effects on pathways inhibiting oxidant production and increasing antioxidant activity.

Biomembranes of tissues are one of the most important targets for free radicals to act on. Free radicals that are not removed by the defense mechanism of the body may induce lipid peroxidation of the membrane, resulting in its dysfunction, such as by gap formation, enzyme inactivation and changing its flowability. van Acker *et al* (41) reported that a glutathione-dependent protective effect was restored when naringenin was injected into α-tocopherol-deficient microsomes, which indicates that flavonoid antioxidants such as naringenin act similarly to α-tocopherol. It was observed that Fe^2+^- and vitamin C-induced lipid peroxidation was inhibited by naringenin via restoration of the protective effect of glutathione in liver microsomes. Jeon *et al* (24) found that naringenin and its metabolites significantly improved SOD and GSH-Px activity in rat liver cytosol. Badary *et al* (42) observed a much higher antioxidant enzyme level and statically improved renal function in rats treated with a combination of naringenin and cisplatin compared with those in rats treated with cisplatin alone in their study, indicating that naringenin reduced cisplatin-induced renal toxicity. Arafa *et al* (43) reported that pretreatment with naringenin was helpful in reducing ADM-induced cardiotoxicity in rats. The current study and the aforementioned related findings provide new inspiration for chemotherapy using exogenous antioxidants in clinical oncology.

In conclusion, the present study revealed the promising value of naringenin in leukemia treatment. Naringenin demonstrated a significant inhibitory effect on the growth of K562 cells, whereas at low concentrations it did not exhibit a cytotoxic effect on normal PMNs. Furthermore, naringenin protected PMNs from oxidative damage by ADM at low concentrations. Cell cycle arrest and the induction of apoptosis, achieved via the p53-independent upregulation of p21/WAF1, a downstream effect of the phosphoinositide 3-kinase (PI3K) pathway, may be the mechanism of the antileukemic effects exhibited by naringenin. The protective effect against the damaging effects of ADM chemotherapy in PMNs may be the result of the antioxidant activity of this agent at its low concentrations. Future studies to elucidate the effects of naringenin and its mechanism are required to further evaluate the value of naringenin in leukemia treatment.

## Figures and Tables

**Table I:** Effects of different concentrations of naringenin on the cell cycle and apoptosis of K562 cells.

	Cell cycle (%)			
		
Concentration (μmol/l)	G_0_/G_1_	S	G_2_/M	Apoptosis rate (%)
Control	41.03±2.80	45.59±1.80	13.38±0.30	2.20±0.30
50	49.96±2.81^a^	34.78±1.72	15.26±1.20	4.02±0.08^b^
400	57.89±2.30^b^	26.12±1.21	15.99±1.61	10.49±2.60^b^
800	59.20±4.70^a^	25.88±3.70	14.92±4.93	20.68±4.91^b^

Values presented are the mean ± standard deviation.

a P<0.05,

b P<0.01 compared with the control group.

The flow cytometry test was performed after 24 h of cell culture.

**Table II:** Effects of naringenin on ADM-induced GSH-Px, SOD, MDA and ROS changes in K562 cells and PMNs.

		K562 cells				PMNs			
			
		Antioxidases		Oxidative stress		Antioxidases		Oxidative stress	
					
Group	Dose (μmol/l)	GSH-Px (μmol/g)	SOD (U/g)	MDA (nmol/g)	ROS (U/g)	GSH-Px (μmol/g)	SOD (U/g)	MDA (nmol/g)	ROS (U/g)
Control	0	361.23±61.04^a^	55.18±7.48^a^	0.46±0.25^a^	80.65±32.12^a^	366.63±42.45^a^	62.36±11.05^a^	0.25±0.08^a^	78.36±27.46^a^
ADM	2	106.76±31.64	9.70±4.34	14.78±3.56	264.74±63.14	158.39±28.36	14.24±2.69	12.37±2.36	226.74±36.76
NGEN	20	265.81±56.23^a^	24.33±3.35^a^	1.45±5.05^a^	94.63±33.51^a^	285.13±35.60^a^	28.93±2.53^a^	1.15±3.12^a^	82.01±35.43^a^
	40	329.56±50.12^a^	30.84±4.75^a^	2.56±3.25^a^	116.42±43.25^a^	342.72±40.23^a^	39.26±4.68^a^	2.42±2.48^a^	93.69±42.48^a^
Post 1 h	20	136.32±43.12	11.56±3.48^b^	11.63±4.32	181.16±25.63	226.12±53.23^a^	21.48±1.65^a^,^c^	2.92±3.37^a^,^c^	102.45±35.14^a^
	40	150.36±46.36^b^	14.48±3.84^b^	13.12±4.45^b^	215.28±32.56	287.56±37.84^a^,^c^	26.94±3.13^a^,^c^	3.08±2.78^a^,^c^	106.56±23.33^a^,^c^
Post 24 h	20	106.79±17.02^b^	10.32±2.53^b^	15.60±6.32^b^	251.85±53.26^b^	145.39±35.23^b^	11.30±2.40^b^	13.29±2.43^b^	211.34±49.79^b^
	40	124.63±40.56^b^	12.64±3.13^b^	16.39±4.25^b^	273.98±43.13^b^	160.74±65.18	13.82±3.45^b^	16.15±2.34^b^	237.35±45.56^b^

Values presented are the mean ± standard deviation.

a P<0.05, compared with the ADM group.

b P<0.05, compared with the naringenin group at the same concentration.

c P<0.05, compared with K562 cells with the same concentration and parameters.

ADM, Adriamycin; GSH-Px, glutathione peroxidase; SOD, superoxide dismutase; MDA, malondialdehyde; ROS, reactive oxygen species; PMN, polymorphonuclear leukocyte; NGEN, naringenin.
